# Long-term response of over ten years with sorafenib monotherapy in metastatic renal cell carcinoma: a case report

**DOI:** 10.1186/s13256-016-0961-0

**Published:** 2016-06-16

**Authors:** Kosuke Ueda, Shigetaka Suekane, Naoyuki Ogasawara, Katsuaki Chikui, Shunsuke Suyama, Makoto Nakiri, Kiyoaki Nishihara, Mitsunori Matsuo, Tsukasa Igawa

**Affiliations:** Department of Urology, Kurume University School of Medicine, 67 Asahi-machi, Kurume, 830-0011 Japan

**Keywords:** Renal cell carcinoma, Long-term response, Sorafenib, Monotherapy

## Abstract

**Background:**

Molecular targeted therapies have dramatically improved the prognosis of metastatic renal cell carcinoma. However, patients in whom the treatment could initially be effective will experience disease progression later.

**Case presentation:**

A 74-year-old Japanese man who was diagnosed with renal cell carcinoma with no evidence of metastasis presented to our hospital. He initially underwent radical nephrectomy, and subsequently the disease metastasized to the lung. Sorafenib was started for the lung metastases 1 year after the operation. The dose of sorafenib was reduced and temporarily discontinued because adverse events, including fatigue and cardiac infarction, occurred. The patient has continued sorafenib monotherapy for over 10 years without disease progression and severe adverse events.

**Conclusions:**

We present a rare case of a patient with metastatic renal cell carcinoma who has survived for over 10 years while receiving sorafenib monotherapy.

## Background

Renal cell carcinoma (RCC) accounts for 3 % of adult malignancies and 90 % of neoplasms arising from the kidney [1]. Approximately 20–40 % of patients with initially localized disease who have had a nephrectomy will go on to develop metastases [2]. Patients with metastatic disease have a poor prognosis, with 5-year survival rates being less than 10 % [2].

In recent years, targeted therapies have dramatically improved the prognosis of patients with metastatic RCC [3–6]. Some groups have demonstrated that there are several prognostic factors for metastatic RCC other than the Memorial Sloan Kettering Cancer Center (MSKCC) risk classification system, including serum C-reactive protein level, metastasis status, and tumor shrinkage [7–10]. Previously, we reported that the duration of first-line treatment with molecular targeted therapies is the strongest prognostic factor in patients with metastatic RCC [11]. In Japan, three types of tyrosine kinase inhibitors (TKIs), such as sorafenib, sunitinib, and pazopanib, and one type of mammalian target of rapamycin are applicable for patients with metastatic RCC as first-line molecular targeted therapy. However, there have been few reports of survival for over 10 years following treatment with only one molecular targeted agent. In this report, we present a case of a patient with metastatic RCC who has survived for over 10 years while receiving sorafenib monotherapy.

## Case presentation

**Fig. 1:**
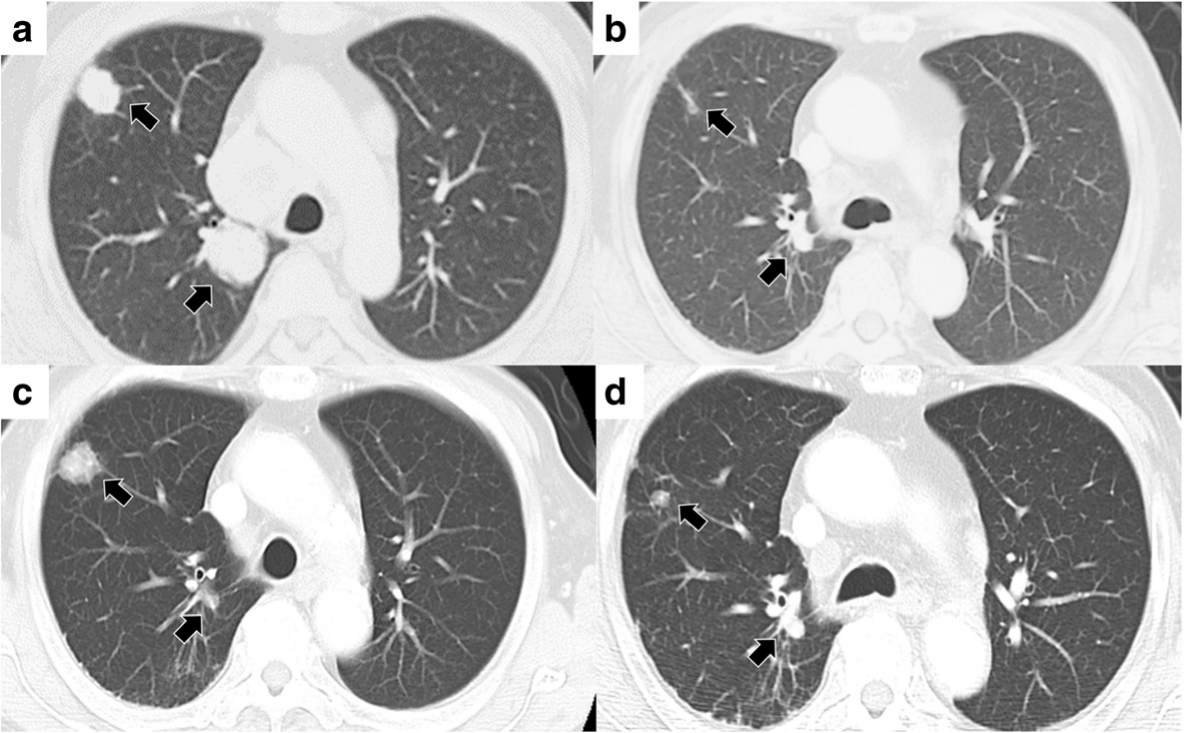
**a** Computed tomographic scan taken before initiation of treatment with sorafenib reveals multiple pulmonary metastatic lesions (*arrows*). **b** Computed tomographic scan obtained 1 year after initiation of sorafenib reveals tumor regression (*arrows*) of 70.1 % according to Response Evaluation Criteria in Solid Tumors. **c** Computed tomographic scan taken 8 months after sorafenib therapy was stopped reveals progression of pulmonary metastases (*arrows*). **d** Pulmonary metastases (*arrows*) remained stable 11 years after initiation of treatment with sorafenib

Our patient was a 74-year-old Japanese man who had been diagnosed with a left renal mass and no evidence of metastasis 11 years before presentation to our hospital. Left radical nephrectomy was performed. The histology of the renal tumor showed clear cell RCC, Fuhrman nuclear grade 2, which was classified as pathological stage T2bN0M0 according to TMN classification. Multiple pulmonary metastases were discovered 10 months after surgery. The patient was stratified as intermediate risk according to the MSKCC criteria. Although immunotherapy (interleukin-2 plus interferon) was started, disease progression was observed on a follow-up computed tomographic (CT) scan 6 months later (Fig. 1a).

Therefore, the patient was included in a phase II clinical trial and was started on treatment with oral sorafenib (Nexavar®; Bayer HealthCare Pharmaceuticals, Whippany, NJ, USA) 800 mg/day 16 months after surgery. Three months after the initiation of sorafenib, a CT scan showed 19.3 % regression based on the Response Evaluation Criteria in Solid Tumors. Then, after 1 year, 70.1 % regression was observed (Fig. 1b). At this time, as grade 2 fatigue and grade 1 aspiration pneumonia emerged, the dose was reduced to 400 mg/day. In addition, the patient twice had acute myocardial infarctions, one 4 years and one 5 years after initiation of sorafenib. Because general fatigue and weakness occurred, sorafenib treatment was stopped 5.5 years after initiation.

Progression of pulmonary metastases was observed 8 months after sorafenib treatment was stopped (Fig. 1c). Although we were reluctant to use a molecular targeted agent, we decided to prescribe sorafenib according to the strong wish of the patient. Treatment with sorafenib was restarted at 200 mg/day. Because this dose was well-tolerated without adverse events, sorafenib was increased to 400 mg/day 5 months after had been restarted. The patient experienced grade 2 fatigue 7.5 years after initiation of sorafenib. Therefore, the schedule was changed to 3 days of treatment and 1 day of rest. The patient has continued sorafenib on this schedule without disease progression or severe adverse events (Fig. 1d).

## Discussion

With the introduction of molecular targeted therapy, treatment of metastatic RCC has improved dramatically. Currently, molecular targeted agents are considered a standard first-line treatment for metastatic RCC. Sorafenib is an oral, multitargeted TKI that targets proliferation and angiogenesis by inhibiting vascular endothelial growth factor receptors 1–3, platelet-derived growth factor receptor (PDGFR)-β, and Raf kinase [3, 12, 13]. In the TARGET study, the median progression-free survival (PFS) and overall survival (OS) of patients treated with sorafenib were 5.5 months and 17.8 months, respectively [3]. Some reports on Japanese patients showed that PFS ranged from 7.4 to 9 months [14, 15]. Although molecular targeted agents such as sorafenib significantly prolong survival of patients with metastatic RCC compared with previous treatment, development of drug resistance is inevitable. Patients in whom treatment is initially effective will eventually experience disease progression. In our patient, cancer control has been achieved for over 10 years with sorafenib monotherapy. Because of adverse events such as fatigue and acute myocardial infarction, the patient temporarily discontinued the treatment. However, since disease progression was observed, the treatment with sorafenib was restarted in accordance with the patient’s wishes.

This case shows long-term survival with use of sorafenib monotherapy. Several biomarkers have been studied as potential predictors of sorafenib response to improve patient selection. Kusuda *et al*. suggested that overexpression of PDGFR-α might be associated with resistance to sorafenib [16]. Jonasch *et al*. demonstrated that a high level of phosphorylated Akt was correlated with inferior PFS and OS in patients treated with sorafenib [17]. In addition, Aziz *et al*. showed that microvessel area size was a predictor of sorafenib response [18]. These biomarkers were not examined in our patient. The utility of molecular biomarkers for predicting clinical outcomes with sorafenib therapy needs to be further studied.

About 3 months after starting and resuming sorafenib therapy, our patient experienced grade 2 fatigue. Also, myocardial infarction occurred twice during treatment with sorafenib. Previous reports suggest that cardiotoxicity is a rare adverse event in patients receiving sorafenib therapy [3, 19]. Schmidinger *et al*. showed that 2.9 % of patients treated with sorafenib experienced treatment-related fatal myocardial infarctions [20]. These events were controlled by dose reduction or discontinuation of sorafenib and symptomatic treatment by cardiologists. As there is the potential for occurrence of occlusion coronary artery disease during sorafenib treatment, patients should be carefully monitored for development of symptoms of cardiac infarction.

There is a question whether long-term treatment with molecular targeted agents increases the incidence of treatment-related adverse events. There are published data analyses of the long-term tolerability of sorafenib in clinical settings. In the phase III study of sorafenib, long-term treatment over approximately 3 years did not increase the overall incidence of treatment-related adverse events [21]. Furthermore, Porta *et al*. showed that long-term treatment with sunitinib did not increase new or more severe treatment-related toxicity [22]. They described how these agents could continue to have clinical benefit without untoward additional risk.

Currently, sequential treatment is strongly recommended for the management of metastatic RCC when disease progression or severe adverse events emerge. Guerin *et al*. reported a case of a patient with metastatic RCC of 14 years’ duration who had a long-term response similar to our patient’s following intermittent therapy with sorafenib [23]. Some researchers have also demonstrated that rechallenge with the same TKI could achieve a tumor response. Some patients with metastatic RCC retain sensitivity to monotherapy [24, 25]. Our present case report suggests that the possible response to sorafenib might be maintained for a long time in a sorafenib responder. In patients with metastatic RCC achieving a continuous response, it is important to continue first-line treatment including sorafenib as long as possible by managing adverse events.

## Conclusions

We present a case of a patient with metastatic RCC with survival for over 10 years with use of sorafenib monotherapy. Biological predictive markers able to identify patients who can sustain durable stabilization of disease are desirable.

## Abbreviations

CT, computed tomography; MSKCC, Memorial Sloan Kettering Cancer Center; OS, overall survival; PDGFR, platelet-derived growth factor receptor; PFS, progression-free survival; RCC, renal cell carcinoma; TKI, tyrosine kinase inhibitor.
